# Targeted Temperature Management for Patients with Acute Ischemic Stroke: A Literature Review

**DOI:** 10.3390/jcm13020586

**Published:** 2024-01-19

**Authors:** Dhanesh D. Binda, Maxwell B. Baker, Shama Varghese, Jennifer Wang, Rafael Badenes, Federico Bilotta, Ala Nozari

**Affiliations:** 1 Department of Anesthesiology, Boston University Chobanian & Avedisian School of Medicine, Boston, MA 02118, USA; ddb96@bu.edu (D.D.B.); maxb98@bu.edu (M.B.B.); shama.varghese@tufts.edu (S.V.); jenrwang@bu.edu (J.W.); ala.nozari@bmc.org (A.N.); 2 Department Anesthesiology, Surgical-Trauma Intensive Care and Pain Clinic, Hospital Clínic Universitari, University of Valencia, 46010 Valencia, Spain; 3 Department of Anaesthesiology, Critical Care and Pain Medicine, Policlinico Umberto I Teaching Hospital, Sapienza University of Rome, 00185 Rome, Italy; bilotta@tiscali.it

**Keywords:** targeted temperature management, acute ischemic stroke, hypothermia, neurocritical care, literature review

## Abstract

Despite significant advances in medical imaging, thrombolytic therapy, and mechanical thrombectomy, acute ischemic strokes (AIS) remain a major cause of mortality and morbidity globally. Targeted temperature management (TTM) has emerged as a potential therapeutic intervention, aiming to mitigate neuronal damage and improve outcomes. This literature review examines the efficacy and challenges of TTM in the context of an AIS. A comprehensive literature search was conducted using databases such as PubMed, Cochrane, Web of Science, and Google Scholar. Studies were selected based on relevance and quality. We identified key factors influencing the effectiveness of TTM such as its timing, depth and duration, and method of application. The review also highlighted challenges associated with TTM, including increased pneumonia rates. The target temperature range was typically between 32 and 36 °C, with the duration of cooling from 24 to 72 h. Early initiation of TTM was associated with better outcomes, with optimal results observed when TTM was started within the first 6 h post-stroke. Emerging evidence indicates that TTM shows considerable potential as an adjunctive treatment for AIS when implemented promptly and with precision, thereby potentially mitigating neuronal damage and enhancing overall patient outcomes. However, its application is complex and requires the careful consideration of various factors.

## 1. Introduction

Acute ischemic stroke (AIS) can be associated with devastating outcomes and considerable mortality, making it the fifth leading cause of death in the United States [1]. Decisions should prioritize salvaging neuronal tissue when approaching treatment goals, with careful attention paid to preventing further cases of ischemia that could lead to irreversible damage within the penumbra. The American Heart Association (AHA) and American Stroke Association (ASA) have updated their guidelines for managing AIS, emphasizing the importance of early intervention [2]. Immediate goals in managing an acute stroke consist of not only minimizing brain injury but also addressing medical complications and unraveling the pathophysiologic basis of the patient’s symptoms to direct the treatment.

Current standards of care for selected patients with large vessel occlusions include both pharmacologic and mechanical interventions, with recombinant tissue-type plasminogen activator (r-tPA) and mechanical thrombectomy recommended per AHA guidelines [2]. Intravenous thrombolysis with r-tPA or tenecteplase, a genetically modified variant with greater fibrin specificity and longer half-life, has been established as an effective treatment for AIS [3]. However, functional outcome is heavily dependent on the timing of administration, with better outcomes reported when the gap between symptom onset and the initiation of intravenous (IV) thrombolysis is reduced [4]. The effectiveness of endovascular thrombectomy is also time-dependent [5]. Greater benefits are observed when thrombectomy is initiated within the first 2 h from symptom onset [5]. Saver et al. (2016) reported that 1.0% of patients experience a more severe disability characterized by a unit increase in the modified Rankin score (mRS) for every 9 min delay from the onset to arterial puncture [5].

While r-tPA and mechanical thrombectomy are both proven therapeutic interventions in treating AIS, other increasingly explored strategies that can help us to delay or reduce a neuronal injury are targeted temperature management (TTM) or the maintenance of a specific core body temperature involving therapeutic hypothermia [6]. Targeted temperature management is thought to protect brain tissue through several mechanisms including a reduction in the cerebral infarction volume, decreased excitotoxicity through downregulated glutamate receptor expression, diminished calcium-dependent signaling, attenuated inflammation through the suppression of astrocyte and microglia activation, and a decrease in metabolism [7]. Similarly to the thrombolytic therapy, TTM’s efficacy is heavily dependent on several factors such as the duration and depth of cooling [8]. Yet, the current body of literature is marred by a substantial disparity in critical factors that can influence the TTM outcome, encompassing variables such as the depth of hypothermia and the duration from the patient’s time last known well to the point of reaching the target temperature. It is therefore difficult to draw conclusions about the efficacy of TTM due to differing evidence from various study designs.

In this review, we aim to highlight specific considerations when administering TTM for AIS, setting the stage for a comprehensive examination of optimal timing and temperature targets.

## 2. Methods

The primary aim of this literature review was to investigate the utility of TTM for stroke patients. This included examining variables such as the target temperature, time to TTM initiation, time to target temperature, duration of TTM, method of cooling, site of the temperature probe, mechanism of stroke, functional neurological outcomes, hospital complications of varying types, and mortality. A comprehensive search of the literature was executed using PubMed, Cochrane, Web of Science, and Google Scholar. Keywords pertinent to the research question were used as follows: targeted temperature management, TTM, stroke, cerebrovascular accident, hypothermia, therapeutic cooling, target temperature, time to TTM initiation, time to target temperature, duration of TTM, method of cooling, site of temperature probe, mechanism of strokes, functional neurological outcomes, and mortality. The search was supplemented by investigating clinical trials, reviews, and guidelines issued by relevant professional associations.

Initial screening involved reviewing titles and abstracts to gauge their applicability to the research question. Articles that met the preliminary criteria were fully analyzed. Preference was given to publications with greater strength of evidence, such as randomized controlled trials, prospective cohort studies, meta-analyses, and noteworthy review articles. Included articles were exclusively written in English; all non-English or off-topic publications were omitted. Each eligible source was systematically studied, noting critical details such as the authors, publication year, research design, sample size, primary results, and conclusions. Data amassed from these sources were methodically categorized into pertinent themes relating to the research question. These classifications were as follows: “Target Temperature”, “Time to TTM Initiation”, “Time to Target Temperature”, “Duration of TTM”, “Method of Cooling”, “Site of Temperature Probe”, “Mechanism of Strokes”, “Functional Neurological Outcomes”, and “Mortality”.

For each thematic category, a synthesis of the literature on the data was conducted. This involved crafting a holistic view of each theme, emphasizing both the consistent findings across different studies and the unique differences. The synthesis followed a strict protocol to ensure an organized and unbiased representation of the available literature on TTM in stroke patients.

## 3. Results

Our search yielded 29 studies that met the inclusion criteria (Table 1 and Table 2). Outcomes including mortality, mRS scores, and rates of pneumonia and hemorrhage in patients were compared by categorizing the studies into two groups as follows: (1) systemic cooling (n = 24, Table 3) and (2) intra-arterial selective cooling infusion (IA-SCI) (n = 5, Table 4). The review identified several key themes regarding target temperature management in patients who experience strokes, including “time to cooling initiation and target temperature”, “method of cooling”, “degree of hypothermia”, “duration of cooling”, “site and mechanism of strokes”, “fibrinolytic use”, and “outcomes.” Figures were generated to illustrate general and specific considerations.

### 3.1. Study Characteristics

#### 3.1.1. Distribution of Study Designs

There was a diverse distribution of study designs in our analysis (Table 1 and Table 2). Among the identified studies, 13 were randomized controlled trials (RCTs) [9,10,11,12,13,14,15,16,17,18,19,20,21], 12 were prospective non-randomized studies [8,22,23,24,25,26,27,28,29,30,31,32], 2 were case–control studies [33,34], 1 was a prospective cohort study [35], and 1 was a prospective observational study [36].

#### 3.1.2. Time to TTM Initiation

The initiation times for TTM varied widely across the systemic cooling studies, ranging from immediately after the stroke to the point exceeding 60 h post-stroke. Among the 24 studies examining time to TTM initiation, 23 reported the times. The most frequent initiation time, as indicated by 39.1% of the studies, occurred less than 6 h after the stroke [9,10,11,14,17,18,27,31,34], which was followed by the initiation time of 12 h after the stroke in 17.4% of the studies [13,26,28,33]. The remaining 43.5% of studies demonstrated variable timeframes for the initiation of TTM ranging from 4.5 to 5 h [19,21] or to 60.0 h [12] (Table 1).

A total of 40.0% of the IA-SCI studies initiated TTM 6 h after the stroke [18,31], 20.0% reported 3 h after the stroke [29], 20.0% began TTM 8 h after the stroke [30], and 20.0% applied TTM 24 h after the stroke [19] (Table 2).

#### 3.1.3. Depth of TTM

Our literature review identified a variety of temperature targets for TTM within 23 of the studies that used systemic cooling. A total of 21.7% of these 23 studies included a target temperature of 32 °C [8,10,12,21,34], 78.2% of the studies had a target temperature of 33 °C [8,10,11,12,13,15,17,21,22,23,25,26,27,28,32,34,35,36], 4.3% of the studies included the temperature of 31 °C in their temperature range [8], 26.1% included a target of 34 °C [9,10,12,20,22,35], and 26.1% included a temperature of 35 °C [9,14,16,20,24,35] in their target temperature for hypothermic cooling.

Studies evaluating IA-SCI reported an insignificant total body temperature change after hypothermic therapy was initiated. All studies utilized continuous perfusion of 4 °C normal saline over an area that had been revascularized.

**Table 1:** Summary characteristics of included systemic cooling studies.

First Author	Year	Study Design	Sample Size	Additional Therapy	Mean Age (Years)	Target Temperature (°C)	Time to TTM Initiation (Hours)	Time to Target Temperature (Hours)	Duration of TTM (Hours)	Method of Cooling	Site of Temperature Probe	Mechanism of Strokes
Schwab [23]	1998	Prospective non- randomized	25	N/A	49.0	33.0	14.0 ± 7.0	3.5–6.2	48.0–72.0	Surface cooling (Polar Bair) with cool ventilator air	Bladder	MCA ischemic stroke
Kammersgaard [33]	2000	Case–control	73 (17 TTM cases)	N/A	68.6	N/A	<12.0 (mean 3.25)	6.0	6.0	Surface cooling (Polar Bair) with cool ventilator air	Tympanic Rectal	Combined ischemic/hemorrhagic
Krieger [34]	2001	Case–control	19 (10 TTM cases)	N/A	71.1	32.0	<6.0	3.5 ± 1.5	22.8 ± 8.0	Surface cooling (Aquamatic K-Thermia EC600 blanket) + alcohol/ice bath	Bladder	MCA ischemic stroke
Schwab [21]	2001	RCT	50	N/A	57.0 ± 8.0	32.0–33.0	22.0 ± 9.0	3.5–11.0	24.0–72.0	Surface cooling	N/A	Cardioembolism (n = 34) ICA dissection with secondary MCA embolization (n = 8) Atherothrombotic disease at the carotid bifurcation (n = 3) Unknown (n = 5)
Georgiadis [32]	2001	Prospective non- randomized	6	Thrombolysis (n = 2)	64.5 ± 8.4	33.0	28.2 ± 17.0	3.0 ± 1.0	67.0 ± 13.0	Endovascular and selective head cooling	Bladder	Acute ischemic stroke
Georgiadis [25]	2002	Prospective non- randomized	19	Hemicraniectomy	56.0	33.0	24.0	4.0 ± 1.0	48.0–72.0	Endovascular OR cold blanket + fan	Thermistor on endovascular catheter	MCA ischemic stroke
Berger [36]	2002	Prospective observational study	12	Antiedema therapy with mannitol	N/A	33.0	<16.3	N/A	48.0–72.0	Systemic surface	N/A	Space-occupying MCA infarction
De Georgia [13]	2004	RCT	40 (18 TTM cases)	N/A	60.9	33.0	<12.0	1.3 ± 0.7	24.0	Endovascular (reprieve endovascular temperature management system)	Esophageal	Anterior circulation territory ischemic stroke
Abou-Chebl [8]	2004	Prospective non- randomized	18	Various	69.6	32.0 ± 1.0	<8.0	3.2 ± 1.5	12.0–72.0	Surface cooling (Aquamatic K-Thermia EC600 blanket) + alcohol/ice bath	Bladder	MCA ischemic stroke
Lyden [26]	2005	Prospective non- randomized	18	N/A	66.2	33.0	<12.0 (mean 3.3)	7.0	12.0 or 24.0	Endovascular (Celsius Control catheter)	Tympanic Bladder Esophageal (2 out of 3)	Acute ischemic stroke
Els [16]	2006	RCT	25 (12 TTM cases)	Hemicraniectomy	49.0	35.0	Immediately after hemicraniectomy	2.0 ± 1.0	48.0	Intravenous saline (Icy, Cool Gard Perfusion Set) Surface cooling (thermo-wrap)	Tympanic Esophageal	Supratentorial ischemic stroke
Guluma [27]	2006	Prospective non- randomized	10	IV thrombolysis	N/A	33.0	<6.0	1.7 ± 0.7	24.0	Endovascular (Celsius Control catheter)	Thermistor on endovascular catheter	N/A
Guluma [28]	2008	Prospective non- randomized	18	IV thrombolysis	64.0	33.0	<12.0	7.7 ± 4.1	12.0 or 24.0	Endovascular (Celsius Control catheter)	Thermistor on endovascular catheter	Acute ischemic stroke
Martin-Schild [22]	2009	Prospective non- randomized	20 (18 TTM cases)	IV thrombolysis (n = 13) Caffeinol (n = 18)	56.0	33.0–34.5	5.0	1.0 (n = 2) 2.0 (n = 4) 3.0 (n = 8) 4 did not reach target	19.8	Endovascular cooling Surface cooling	Bladder	Acute ischemic stroke
Hemmen [11]	2010	RCT	59 (28 TTM cases)	IV thrombolysis	65.5	33.0	<6.0	Stratified 0.0–3.0 3.0–6.0	24.0	Endovascular (Celsius Control Catheter)	Thermistor on endovascular catheter	Acute ischemic stroke
Bi [10]	2011	RCT	93 (31 TTM cases)	IV thrombolysis	Group A: 68.5 ± 6.9	32.0–34.0	<6.0	0.3	24.0	Surface Cooling	Rectal	Acute ischemic stroke
Hong [35]	2014	Prospective cohort study	75 (39 TTM cases)	IV thrombolysis	64.5 ± 17.0	34.4 ± 0.9	N/A	6.3 ± 5.9	48.0	Endovascular Surface cooling	Esophageal	Acute ischemic stroke involving the anterior circulation
Ovesen [15]	2013	RCT	31 (17 TTM cases)	IV thrombolysis	62.3	33.0	<24.0	14.9	24.0	Endovascular Surface cooling	Bladder	Acute ischemic stroke
Piironen [14]	2014	RCT	36 (18 TTM cases)	IV thrombolysis	68.0	35.0	<6.0	6.0 (4.5–6.5)	12.0	Intravenous Saline, then maintained by surface cooling	Bladder	Acute ischemic stroke
Lyden [17]	2016	RCT	120 (63 TTM cases)	IV thrombolysis	65.5 ± 10.3	33.0	<6.0	4.8 ± 1.1	24.0	Endovascular (Celsius Control Catheter)	N/A	Acute ischemic stroke
Geurts [20]	2017	RCT	22 (16 TTM cases)	N/A	63.0	34.0 34.5 35.0	<4.5	6.8 (34.5 °C) 7.4 (35.0 °C)	24.0	Intravenous saline + surface cooling	N/A	Acute ischemic stroke
van der Worp [9]	2019	RCT	98 (49 TTM cases)	N/A	69.6	34.0–35.0	<6.0	N/A	12.0 or 24.0	IV saline Surface cooling	Rectal Bladder	N/A
Neugebauer [12]	2019	RCT	50 (26 TTM cases)	Hemicraniectomy	51.3 (excluded over 60)	33.0 ± 1.0	<60.0	N/A	72.0	Endovascular Surface cooling	Bladder	MCA
Bardutzky [24]	2023	Prospective non- randomized	22	Various	77.0	35.0	After admission: 1.0 (0.7–1.3) After EVT-dependent intubation: 0.08 (0.06–0.09) Prior to groin puncture: 0.09 (0.03–0.17)	0.5 (0.4–0.6)	6.0 (post-recanalization)	Transnasal (RhinoChill)	Esophageal Tympanic	Occlusion of the M1 or M2 segment of the MCA or ICA or tandem occlusion; ischemic stroke

Note: N/A (not available), TTM (targeted temperature management), RCT (randomized controlled trials), IV (intravenous), EVT (endovascular treatment), ICA (internal carotid artery), MCA (middle cerebral artery).

**Table 2:** Summary characteristics of included intra-arterial selective cooling studies.

First Author	Year of Publication	Study Design	Sample Size	Additional Therapy	Mean Age (Years)	Target Temperature (°C)	Time to TTM Initiation (Hours)	Time to Target Temperature (Minutes)	Duration of TTM (Minutes)	Method of Cooling	Site of Temperature Probe	Mechanism of Strokes
Kollmar [29]	2009	Prospective non- randomized	10	IV thrombolysis	66.5 ± 12.5	N/A	<3.0	N/A	240.0	Intra-arterial Selective cooling infusion	Tympanic	Acute ischemic stroke
Chen [30]	2016	Prospective non- randomized	26	Recanalization (various)	58.4	N/A	<8.0	5.0 (pre-stent)	10.0 (post-stent)	Intra-arterial Selective cooling infusion	Rectal	Proximal vessel (ICA, MCA M1 + M2, BA/VA) ischemic stroke
Peng [18]	2016	RCT	26 (11 TTM cases)	IV thrombolysis	N/A	N/A	<6.0	N/A	10.0	Intra-arterial Selective cooling infusion	N/A	MCA
Wu [31]	2018	Prospective non- randomized	113 (45 TTM cases)	Mechanical thrombectomy	62.1	N/A	<6.0	N/A	15.0	Intra-arterial Selective cooling infusion	N/A	M1 segment of the MCA
Wan [19]	2023	RCT	142 (71 TTM cases)	Mechanical thrombectomy	73.4	N/A	<24.0	N/A	35.0	Intra-arterial Selective cooling infusion	N/A	M1 and M2 segments of the ICA and MCA, including the extracranial and intracranial segments

Note: N/A (not available), TTM (targeted temperature management), RCT (randomized controlled trials), IV (intravenous), ICA (internal carotid artery), MCA (middle cerebral artery).

**Table 3:** Summary outcomes of included systemic cooling studies.

		Normothermic							Hypothermic						
		Stroke			Health Outcomes				Stroke			Health Outcomes			
First Author	Year	Severity (NIHSS)	Functionally Independent (%)	Infarct Volume (mL)	Mortality (%)	Pneumonia (%)	Edema Formation (%)	Hemorrhage (%)	Severity (NIHSS)	Functionally Independent (%)	Infarct Volume (mL)	Mortality (%)	Pneumonia (%)	Edema Formation (%)	Hemorrhage (%)
Schwab [23]	1998	N/A	N/A	N/A	78.0	N/A	N/A	N/A	4 weeks: 29.0 (25.0–37.0) 3 months: 38.0 (28.0–48.0)	N/A	N/A	44.0	40.0	N/A	N/A
Kammersgaard [33]	2000	6 months: 47.9 ± 11.4	N/A	N/A	28 days: 11.0 6 months: 23.0	13.0	N/A	N/A	6 months: 42.4 ± 13.7	N/A	N/A	28 days: 6.0 6 months: 12.0	18.0	N/A	N/A
Krieger [34]	2001	Baseline: 19.6 ± 2.6	3 months: 11.1	N/A	3 months: 22.2	11.1	N/A	N/A	Baseline: 19.8 ± 3.3	3 months: 50.0	N/A	3 months: 30.0	30.0	N/A	N/A
Schwab [21]	2001	N/A	N/A	N/A	N/A	N/A	N/A	N/A	Baseline: 25.0 (15.0–32.0) 4 weeks: 29.0	N/A	N/A	38.0	48.0	N/A	N/A
Georgiadis [32]	2001	N/A	N/A	N/A	N/A	N/A	N/A	N/A	N/A	N/A	N/A	16.7	100.0	N/A	N/A
Georgiadis [25]	2002	Baseline: 17.0 (16.0–18.0)	N/A	N/A	18 months: 12.0	N/A	N/A	N/A	Baseline: 20.0 (18.0–22.0)	N/A	N/A	18 months: 47.0	18 months: 78.9	N/A	N/A
Berger [36]	2002	N/A	N/A	N/A	N/A	N/A	N/A	N/A	N/A	N/A	N/A	33.3	N/A	N/A	N/A
De Georgia [13]	2004	Baseline: 14.6 ± 5.6 Baseline corrected: 16.7 ± 4.4	N/A	N/A	10	5.0	2.5	N/A	Baseline: 15.2 ± 4.4 Baseline corrected: 18.2 ± 4.4	N/A	Infarct volume growth was less in the hypothermia group but not significant	12.5	5.0	7.5	2.5
Abou-Chebl [8]	2004	N/A	N/A	N/A	N/A	N/A	N/A	N/A	Baseline: 21.4 ± 5.6	N/A	N/A	11.1	27.8	N/A	5.6
Lyden [26]	2005	N/A	N/A	N/A	N/A	N/A	N/A	N/A	N/A	N/A	N/A	16.7	30 days: 5.6	N/A	16.7
Els [16]	2006	Baseline: 19.0 ± 2.0 6 months: 10.0 ± 1.0	N/A	N/A	15.0	N/A	N/A	N/A	Baseline: 18.0 ± 2.0 6 months: 11.0 ± 3.0	N/A	N/A	8.0	N/A	N/A	N/A
Guluma [27]	2006	N/A	N/A	N/A	N/A	N/A	N/A	N/A	N/A	N/A	N/A	0.0	0.0	0.0	0.0
Guluma [28]	2008	Immediately following catheter removal: 12.3 ± 8.5 30 days: 9.1 ± 7.8	N/A	30 days: 73.0 ± 71.0	N/A	N/A	N/A	N/A	Immediately following catheter removal: 13.3 ± 11.0 30 days: 14.4 ± 14.3	30 days: no difference in modified Rankin scores	30 days: 84.0 ± 102.0	N/A	N/A	Significantly decreased	N/A
Martin-Schild [22]	2009	N/A	N/A	N/A	N/A	N/A	N/A	N/A	Baseline: 15.0 24 h: 9.0 Discharge/5 days: 6.0	Discharge: 40.0	N/A	Hospitalization: 15.0	20.0	1.0	20.0
Hemmen [11]	2010	Baseline: 13.7 ± 5.1 24 h: 11.1 ± 8.1 1 month: 5.0 ± 4.1 3 months: 3.8 ± 3.0	90 days: 24.0	N/A	90 days: 16.7	10.0	N/A	48 h: 25.0	Baseline: 14.3 ± 5.0 24 h: 17.0 ± 8.9 1 month: 8.0 ± 6.5 3 months: 6.3 ± 6.6	90 days: 18.0	N/A	90 days: 21.4	50.0	N/A	48 h: 33.0
Bi [10]	2011	Thrombolysis baseline: 11.0 ± 2.7 Thrombolysis 90 days: −4.1 ± 0.5 Anti-platelet baseline: 10.8 ± 2.7 Anti-platelet 90 days: −1.5 ± 0.4	90 days: thrombolysis 39.3 anti-platelet 13.8	N/A	90 days: thrombolysis 9.7 anti-platelet 6.5	N/A	N/A	24 h: thrombolysis symptomatic 3.2 thrombolysis Asymptomatic 19.4 Anti-platelet symptomatic 0.0 Anti-platelet asymptomatic 6.5	Baseline: 11.4 ± 2.8 90 days: −4.0 ± 0.6	90 days: 48.1	N/A	90 days: 12.9	N/A	N/A	24 h: symptomatic 6.5 asymptomatic 16.1
Hong [35]	2014	Baseline: 15.5 (12.0–17.0)	90 days: 22.2	N/A	1 month: 13.9	30.6	83.3	86.1	Baseline: 17.0 (15.0–18.0)	90 days: 48.7	N/A	1 month: 15.4	5.1	46.2	61.5
Ovesen [15]	2013	Baseline: 9.0 48 h: 6.0 7 days: 6.0 90 days: 4.0	N/A	N/A	9.0	9.0	N/A	14.3	Baseline: 8.0 48 h: 15.0 7 days: 11.0 90 days: 4.0	N/A	N/A	12.0	35.0	N/A	5.9
Piironen [14]	2014	Baseline: 14.0	3 months: 39.0	N/A	N/A	3 days: 11.0	3 days: 44.0	3 days: 28.0	Baseline: 11.0	3 months: 39.0	N/A	0.0	3 days: 39.0	3 days: 28.0	3 days: 33.0
Lyden [17]	2016	Baseline: 14.5 ± 4.9 7 days: 10.6 ± 11.3	90 days: 38.0	N/A	8.8	10.5	N/A	3.5	Baseline: 14.1 ± 4.8 7 days: 10.4 ± 10.3	90 days: 33.0	N/A	15.9	19.0	N/A	1.6
Geurts [20]	2017	Baseline: 13.0 3 months: 4.0	3 months: 50.0	N/A	3 months: 17.0	0.0	N/A	0.0	Baseline: 13.0 3 months: 8.0	3 months: 37.0	N/A	3 months: 12.0	53.0	N/A	13.3
van der Worp [9]	2019	Baseline: 11.0 (7.0–17.0) 3 months: 3.0 (1.0–11.0)	3 months: 39.0	N/A	3 months: 8.2	4.1	2.0	4.1	Baseline: 11.0 (7.0–17.0) 3 months: 3.0 (1.0–8.0)	3 months: 50.0	34.3 (10.5–65.5)	3 months: 10.2	18.4	8.2	8.2
Neugebauer [12]	2019	Baseline: 20.5 (15.0–42.0) 14 days: 22.0 (16.0–33.0)	12 months: 4.0	N/A	14 days: 12.5 12 months: 13.0	63.0	N/A	N/A	Baseline: 21.0 (15.0–42.0) 14 days: 25.0 (17.0–37.0)	12 months: 0.0	N/A	14 days: 19.2 12 months: 24.0	54.0	N/A	N/A
Bardutzky [24]	2023	N/A	N/A	N/A	N/A	N/A	N/A	N/A	Baseline: 15.0 (12.5–19.8) 24 h: 7.0 (3.0–12.5) 7 days: 2.0 (1.0–8.0)	Discharge: 64.0 3 months: 68.0	N/A	3 months: 9.0	18.0	N/A	18.0

Note: N/A (not available), NIHSS (national institutes of health stroke scale).

**Table 4:** Summary outcomes of intra-arterial selective cooling studies.

		Normothermic							Hypothermic						
		Stroke			Health Outcomes				Stroke			Health Outcomes			
First Author	Year	Severity (NIHSS)	Functionally Independent (%)	Infarct Volume (mL)	Mortality (%)	Pneumonia (%)	Edema Formation (%)	Hemorrhage (%)	Severity (NIHSS)	Functionally Independent (%)	Infarct Volume (mL)	Mortality (%)	Pneumonia (%)	Edema Formation (%)	Hemorrhage (%)
Kollmar [29]	2009	N/A	N/A	N/A	N/A	N/A	N/A	N/A	Baseline: 5.5 (4.0–12.0) 24 h: 3.0 (1.0–13.0)	N/A	N/A	N/A	N/A	N/A	N/A
Chen [30]	2016	N/A	N/A	N/A	N/A	N/A	N/A	N/A	N/A	N/A	N/A	N/A	38.5	N/A	0.0
Peng [18]	2016	Baseline: 16.8 ± 8.2 24 h: 12.3 ± 5.5 1 month: 9.2 ± 3.4	N/A	Baseline: 25.0 ± 10.1 24 h: 25.6 ± 10.2 7 days: 26.4 ± 10.9	N/A	N/A	N/A	N/A	Baseline: 16.3 ± 8.5 24 h: 10.0 ± 6.9 1 month: 7.1 ± 5.1	N/A	Baseline: 24.9 ± 9.4 24 h: 13.5 ± 6.0 7 days: 12.3 ± 7.4	N/A	N/A	N/A	N/A
Wu [31]	2018	Baseline: 16.0 (11.0–19.0)	90 days: 41.2	3–7 days: 77.9 ± 44.7	90 days: 26.5	7 days or discharge: 33.8	N/A	7 days or discharge: 32.4	Baseline: 17.0 (13.0–21.0)	90 days: 51.1	3–7 days: 63.7 ± 31.8	90 days: 20.0	7 days or discharge: 31.1	N/A	7 days or discharge: 35.6
Wan [19]	2023	Baseline: 16.0 ± 8.0 14 days: 3.5 ± 2.1	90 days: 35.2	7 days: 88.5 ± 20.8	90 days: 8.5	7 days: 21.1	N/A	24 h: 15.5	Baseline: 15.0 ± 7.0 14 days: 2.0 ± 1.2	90 days: 54.9	7 days: 63.7 ± 22.1	90 days: 7.0	7 days: 23.9	N/A	24 h: 18.3

Note: N/A (not available), NIHSS (national institutes of health stroke scale).

#### 3.1.4. Duration of TTM

Targeted temperature management was initiated for various durations (Table 1). Out of 24 systemic cooling studies providing a time duration, 12.5% investigated TTM duration for 12 or 24 h [9,26,28], 29.2% focused on a 24 h duration [10,11,13,15,17,20,27], while 8.3% explored a 48 h timeframe [16,35]. Additionally, 8.3% used a 6 h TTM duration [24,33], and 4.2% applied TTM for 72 h [12]. Furthermore, 4.2% aimed for a 12 h TTM duration [14], 4.2% reported a duration of 19 h and 48 min [22], 4.2% used TTM from 12 h to 72 h [8], 4.2% considered an extended timeframe of 24 to 72 h [21], and 12.5% included a range of 48 to 72 h [23,25,36]. Notably, 4.2% detailed a TTM duration of 67 ± 13 h [32], and 4.2% maintained target temperature for 22.8 ± 8 h [34].

Studies examining IA-SCI reported considerably shorter periods of hypothermia compared with those utilized in systemic cooling (Table 2). The longest duration of TTM reported was 4 h [29]. Other studies reported cooling times of 35 min [19] and 15 min [31], with two remaining studies utilizing a cold saline perfusion duration of 10 min [18,30].

#### 3.1.5. Fibrinolytic Therapies

A total of 54.1% of the 24 systemic cooling studies used thrombolysis in addition to TTM [8,10,11,14,15,17,22,24,27,28,32,35] (Table 1). As for the five IA-SC studies, 40.0% incorporated thrombolysis therapies with TTM [18,29].

### 3.2. Outcomes

#### 3.2.1. Mortality Rates

For systemic cooling, 95.8% of the studies provided data on mortality, revealing a range from 0.0% to 47.0% within the hypothermic groups [8,9,10,11,12,13,14,15,16,17,20,21,22,23,24,25,26,27,32,33,34,35,36]. In comparison, the mortality for normothermic participants was reported in 58.3% of the studies, demonstrating a rate from 6.5% to 78.0% [9,10,11,12,13,15,16,17,20,23,25,33,34,35].

Of the IA-SCI studies, 40.0% reported patient survival rates. On average, patients who remained normothermic had a mortality of 8.5% to 26.5%, whereas patients treated with hypothermia experienced mortality rates of 7.0% to 20.0% [19,31].

#### 3.2.2. NIHSS Severity Changes

Among 15 studies reporting on normothermic participants, 53.3% indicated a decrease in national institutes of health stroke scale (NIHSS) severity [9,10,11,15,16,17,20,28], while 6.7% indicated an increase in NIHSS severity [12]. For the 19 studies reporting the use of hypothermic systemic cooling, 47.4% reported a decrease in NIHSS severity after TTM [9,10,11,15,16,17,20,22,24], while 21.1% reported an increase in NIHSS severity [12,21,23,28]. Notably, Hemmen et al. (2010) observed that the NIHSS value increased 24 h post-TTM and subsequently declined between one and three months [11]. Conversely, Ovesen et al. (2013) reported an increase in NIHSS 48 h post-TTM and at 7 days post-intervention, with a subsequent decline observed after 90 days [15].

Three studies examining IA-SCI reported a decrease in NIHSS scores in patients who had been treated with TTM compared with those who had not been treated with hypothermia [18,19,29]. Peng et al. (2016) reported a statistically significant difference in the 1 -month NIHSS scores among patients who had been treated with TTM compared with those who had remained normothermic [18].

#### 3.2.3. Functional Outcomes

The percentage of patients who were functionally independent (with mRS scores between 0 and 2) and normothermic ranged from 4.0% to 50.0% at 3 to 12 months post-treatment. Of the 9 studies that recorded normothermic values, 22.2% reported that 0.0% to 9.0% of patients were functionally independent [12,35]. Additionally, 22.2% of the studies documented functional independence among 10.0% to 19.0% of patients [10,34], 11.1% of the studies reported that 20.0% to 29.0% of individuals demonstrated functional independence [11], and 55.6% of the studies reported that a 30.0% or higher percentage of their participants had received a mRS score from 0 to 2 [9,10,14,17,20].

Among hypothermic patients who were systemically cooled, 11 studies reported functional independence among a percentage of patients ranging from 0.0% to 68.0% [9,10,11,12,14,17,20,22,24,34,35]. A total of 9.1% of these 11 studies reported that 0.0% of patients achieved mRS scores within the functional independence range [11]. In 9.1% of these studies, 10.0% to 19.0% of patients were functionally independent [10]. The remaining 81.8% reported at least 30.0% of their patients reaching functional independence post-TTM [9,10,14,17,20,22,24,34,35].

Two studies examining IA-SCI reported post-treatment mRS scores at 90 days [19,31]. Patients who were functionally independent in the normothermic group ranged from 35.2% to 41.2% [19,31]. Functionally independent patients in the IA-SCI group were between 51.1% and 54.9% [19,31]. Wan et al. (2023) reported that those in the IA-SCI group were found to have a statistically significant improvement in functionally independent mRS scores compared with those who did not receive IA-SCI [19].

#### 3.2.4. Pneumonia Rates

Normothermic patients who developed pneumonia as a complication were reported in 11 studies, with rates ranging from 0.0% to 63.0% [9,11,12,13,14,15,17,20,33,34,35]. A total of 36.4% of these 11 studies reported pneumonia in up to 10.0% of patients [9,13,15,20], 45.5% of these studies reported the incidence to lie between 10.0% and 20.0% [11,14,17,32,33] and 18.2% of these studies documented pneumonia rates surpassing 30.0% [12,35]. Among the patients who were cooled systemically in 20 studies, between 0.0% and 100.0% developed pneumonia (Table 3 and Table 4). Pneumonia rates were less than 9.9% in 20.0% of these studies [13,26,27,35], while 30.0% of these studies reported pneumonia rates from 10.0% to 29.9% [8,9,17,22,24,33], and 50.0% of the studies indicated pneumonia rates exceeding 30.0% [11,12,14,15,20,21,23,25,32,34].

Pneumonia rates were recorded in 60.0% of the studies examining the use of IA-SCI. A total of between 21.1% and 33.8% of the patients who remained normothermic within IA-SCI studies developed pneumonia [19,31]. Patients treated with IA-SCI experienced comparable pneumonia rates of between 23.9% and 38.5% [19,30,31].

#### 3.2.5. Hemorrhage Rates

A total of 37.5% of the 24 studies that used systematic cooling methods documented the incidence of a hemorrhage as a complication in normothermic patients, which ranged from 0.0% to 86.1% of the patients [9,10,11,14,15,16,17,20,35]. Among the hypothermic treatment group, hemorrhage rates were reported in 58.3% of the studies, with an incidence from 0.0% to 61.5% [8,9,10,11,13,14,15,17,20,22,24,26,27,35].

For studies using IA-SCI, hemorrhage rates post-operation were reported in 60.0% of them. Patients who remained normothermic experienced hemorrhage rates ranging from 15.5% to 32.4% [19,31]. Patients treated with IA-SCI experienced hemorrhaging rates between 0.0% and 35.6% [19,30,31].

## 4. Discussion

### 4.1. Mechanism of TTM for Stroke Management

Stroke remains a leading cause of morbidity and mortality worldwide as many patients presented to the hospital with AIS do not arrive within the therapeutic window for thrombolysis [37]. In our review, TTM was predominately utilized in patients suffering from ischemic strokes. Studies have demonstrated that TTM, when initiated in a timely manner, can reduce infarct size by approximately 44.0% and improve neurological outcomes [6]. In the context of hemorrhagic strokes, which carry a higher mortality rate than ischemic strokes, TTM has been shown to reduce the disruption of the blood–brain barrier and perihematomal edema, although these benefits have not correlated with improved neurological outcomes. Targeted temperature management has not been associated with a reduction in the size of the initial lesion in hemorrhagic strokes but may reduce the extent of an edema during the first seven days [6]. Subarachnoid hemorrhages (SAHs) pose a unique scenario where hyperthermia can worsen neurological outcomes. Temperature control in these patients is associated with a reduced risk of poor neurological outcomes. However, the benefits of TTM for SAHs have not been conclusively proven, with some studies reporting increased rates of bacteremia without clinical benefits [6].

The subacute stage of a stroke, generally considered to be from 1 to 7 days post-ischemia, is of particular importance when implementing therapies that are aimed at mitigating secondary injury, including blood–brain barrier disruption, edema formation, and hemorrhagic transformation. Targeted temperature management has been shown to prevent the accumulation of excitotoxic amino acids, such as glutamate, and to preserve the tissue ATP levels necessary for the maintenance of ion gradients and the prevention of a calcium influx [38,39]. Moreover, TTM impacts early molecular events like the induction of an immediate early gene expression and the cellular stress response, which include the upregulation of the neuroprotective protein HSP70 under hypothermic conditions [40]. MicroRNAs (miRNAs) and cold-inducible proteins such as CIRBP and RBM3, which are implicated in the neuroprotective mechanisms of hypothermia, are also affected through TTM. These molecules are essential in stroke pathogenesis and neuroprotection [41,42]. Targeted temperature management affects multiple cell death and survival pathways during this period [43]. Additionally, TTM is shown to modulate the post-ischemic inflammatory response. It has been shown to lower the numbers of infiltrating neutrophils, activated microglia, and inflammatory mediators such as ROS and proinflammatory cytokines [44,45]. In the chronic phase of strokes, TTM may have long-lasting effects on recovery and repair mechanisms, thus impacting processes such as neurogenesis and synaptogenesis [46,47].

### 4.2. Time to Cooling Initiation and Target Temperature

The initiation times for TTM in our review ranged from immediately to more than 60 h after the stroke. The time from the patient’s time last known well to the initiation of cooling is a pivotal factor in the effectiveness of TTM. Delays can significantly impact patient outcomes as patients who reach the target temperature more quickly have better prognosis than those who took longer [48]. The Neuroprotective Therapy Consensus Review (NTCR) guideline recommends that after intracerebral hemorrhages, subarachnoid hemorrhages, and AIS in patients who require critical care admission, TTM should begin within 1 h of the first fever identification, and shivering managed to limit the risk of secondary injury [49].

Regarding the target temperature of TTM, maintaining the patient’s core temperature between 36.0 °C and 37.5 °C is important, with an ideal central temperature being 36.5 °C, to allow for variability in measurement techniques and device accuracy [49]. Managing brain temperature carefully is critical since it can be up to 2 °C higher than the core temperature and exceeding 37.5 °C could be detrimental. Precision in temperature control is vital, with the goal being to limit temperature variation to less than ±0.5 °C per hour and less than 1 °C over a 24 h period in order to optimize patient outcomes during TTM. The precise control of temperature during TTM is a complex but essential component of care for patients with AIS as it can have a significant impact on the effectiveness of the treatment and the patient’s recovery.

### 4.3. Method of Cooling

Different methods of inducing hypothermia have been explored in the studies included in our review, such as pharmacologic agents, surface cooling devices, endovascular cooling catheters, selective external head cooling, nasopharyngeal brain cooling, and regional intra-arterial cold saline infusion. The choice of method often depends on the balance between efficacy and practical considerations. For instance, antipyretic medications are generally recommended for fever management in acute stroke patients, but their efficacy in normothermic patients remains unproven. High-dose acetaminophen, however, has shown a modest ability (~0.3–0.4 °C) to reduce body temperature even in normothermic individuals [50]. Surface cooling methods may include a cooling blanket combined with ice water and alcohol baths. However, issues such as patient shivering, imprecise temperature control, the time lag to reach target temperature, and rebound hyperthermia can arise with surface cooling techniques, particularly when used in intubated, pharmacologically paralyzed patients [51]. Endovascular cooling offers rapid induction and tight temperature control, with reduced shivering, but it is more invasive and carries risks such as bleeding and thrombosis [52]. Selective external head cooling and nasopharyngeal cooling are less invasive and may be initiated by paramedics, offering localized cooling [53]. Additionally, the use of refrigerated intravenous fluids and gastric lavage with iced fluids has been recommended to accelerate the cooling process [54]. Lavinio et al. (2023) emphasize the use of an automated device to maintain this normothermia, which is particularly important in a critical care setting for patients with intracerebral hemorrhage, aneurysmal subarachnoid hemorrhage, or AIS [49].

The most common method for monitoring temperature during hypothermia studies is the use of a bladder probe. This involves measuring core body temperature with a Foley catheter equipped with a temperature sensor. However, various other sites are used, including tympanic, rectal, pulmonary artery, esophageal, skull surface, and intraparenchymal locations, depending on the study design and practicality. Our review indicates that while the target temperature is critical, the methods to achieve, measure, and maintain this temperature are equally important and should be carefully chosen based on the patient’s condition and the healthcare setting. Continued investigation into these methods, alongside considerations of efficacy, safety, and practicality, will be important in optimizing the application of therapeutic hypothermia in clinical settings for the treatment of AIS.

### 4.4. Degree of Hypothermia

The degree of hypothermia achieved in therapeutic interventions can be categorized as mild (33–36 °C), moderate (28–33 °C), and severe (below 28 °C). While mortality rates ranged from 0% to 47% in our review, most studies reported a mortality rate from 10% to 20% using temperatures from 32 °C to 35 °C. Although there are no established guidelines for the use of therapeutic hypothermia in stroke patients [53], clinical studies of TTM have generally used mild or moderate hypothermia for neuroprotection and reducing metabolic demand. This range is often optimal due to the increased incidence of side effects such as hypokalemia, cardiac rhythm disturbances, infectious complications, and coagulopathy associated with lower temperatures [55]. In addition, severe hypothermia typically requires sedation and mechanical ventilation, complicating the assessment of neurological status and increasing the risk of adverse events [55]. The impact of hypothermia on infections, particularly pneumonia, is a critical aspect of patient care during hypothermia treatment. Given the immunosuppressive effects of stroke [56], combined with hypothermia, there is an increased susceptibility to infections, which might necessitate the use of prophylactic antibiotic treatment during hypothermia in stroke patients [55].

### 4.5. Duration of Cooling

The duration of TTM in the treatment of AIS is variable and depends largely on the protocol of the individual study. The duration of TTM in our review ranged from 6 to 72 h. The optimal duration, however, is not definitively established, and ongoing research aims to ascertain whether shorter or longer periods yield more benefit. Andresen et al. (2015) demonstrated that prolonged hypothermia (over 5 days) has been more effective than conventional hypothermia (around 2 days) in reducing poor neurological outcomes in patients with severe TBI [6]. In the treatment of cardiac arrest, the TTM trial [57] used a 24 h period of hypothermia, which showed benefits in terms of survival and neurological outcomes. Neugebauer et al. (2013) discussed that in the context of malignant middle cerebral artery (MCA) infarction, most patients underwent TTM for a period of one to three days, while only a few smaller studies explored longer durations up to a maximum of 22 days [58]. In an RCT investigating hypothermia in addition to hemicraniectomy for reduction of mortality in patients with malignant MCA stroke, prolonging TTM to 48 h did not significantly improve neurologic outcomes compared with a standard 24 h period [12]. However, it did increase the likelihood of adverse events. This indicates a need for a balance between the potential benefits and risks of prolonged TTM, highlighting the importance of considering the duration of hypothermia treatment carefully in clinical practice. Additionally, there may be synergistic effects when TTM is combined with other stroke therapies, such as decompressive hemicraniectomy, which may influence the duration of cooling required [59]. As such, determining the appropriate duration of TH may also depend on the concomitant treatments being used and the individual patient’s condition. In ischemic stroke, specifically, the optimal duration of TTM remains undetermined. This uncertainty is partly due to the variability in stroke severity, the affected brain regions, and individual patient factors. The lack of a systematic investigation in clinical studies underscore the need for more research to establish evidence-based protocols for TTM in stroke patients. These studies will likely consider not just the duration of cooling but also the depth of hypothermia and the patient’s response to treatment, potentially using serum biomarkers or MRI imaging to monitor the effect of TTM on lesion growth and patient recovery [60,61].

### 4.6. Fibrinolytic Therapies

Almost half of the studies that we reviewed incorporated thrombolysis therapies in addition to TTM. In the intravenous thrombolysis plus hypothermia for acute treatment of ischemic stroke (ICTuS-L) study, the approach of combining endovascular cooling with thrombolysis was assessed for its feasibility and preliminary safety [11]. Patients in this multicenter trial received standard dosed r-tPA and underwent 24 h of endovascular cooling at 33 °C followed by 12 h of controlled rewarming. The ICTuS-L trial did not show a significant benefit in mortality or functional outcomes for patients treated with hypothermia [11]. The ICTuS-L trial, however, established the groundwork for further research, with subsequent trials like ICTuS 2 and ICTuS 3 designed to test the safety and efficacy of combined thrombolysis and endovascular hypothermia more rigorously. The trial also underscored the importance of monitoring for pneumonia and provided insights into the management of hypothermia in acute stroke patients, particularly when combined with thrombolytic therapy. The COOL AID Oresund study investigated endovascular cooling versus surface cooling strategies in the treatment of acute strokes [15]. This bicenter randomized trial included patients with persisting deficits at 3 h after thrombolysis. Among the patients who underwent TTM, 45.0% received thrombolysis, and mortality rates were comparable between the two groups.

### 4.7. Comorbidities

Comorbidities are known to play a significant role in both the treatment decisions and outcomes of stroke patients [62]. Conditions such as hypertension, diabetes, hyperlipidemia, smoking, and atrial fibrillation are all factors that can complicate the management of AISs and influence the effectiveness of treatments like thrombolysis and hypothermia. For instance, hypertension may affect the decision to administer r-tPA and can increase the risk of hemorrhagic transformation post-thrombolysis. Diabetes and hyperlipidemia are linked with atherosclerosis, which can complicate mechanical thrombectomy procedures [63,64]. Smoking is a well-known risk factor for vascular diseases and can negatively impact recovery [65]. Atrial fibrillation is associated with a higher risk of recurrent strokes and may necessitate anticoagulation therapy, which has to be balanced with the risks of thrombolytic therapy [66]. Neugebauer et al. (2019)’s study participants had several comorbidities that are common in the stroke population, including arterial hypertension, diabetes mellitus, hyperlipidemia, habitual smoking, and atrial fibrillation [12]. The COOL AID study also provided insights into comorbidities [33]. The presence of chronic conditions like atrial fibrillation was noted to affect the hypothermia process and patient outcomes. This indicates that patient comorbidities can influence the efficacy and safety of hypothermia treatment, and these factors must be considered when interpreting the results of such interventions [33]. The interplay of these comorbidities with stroke treatment modalities is complex, and a thorough understanding of each patient’s unique medical history is essential for optimizing treatment strategies. Further research from broader clinical studies would be beneficial to the deepening of the understanding of how these comorbidities affect the efficacy of stroke treatments and to the development of guidelines that manage strokes in patients with multiple comorbid conditions.

### 4.8. Outcomes

**Figure 1:**
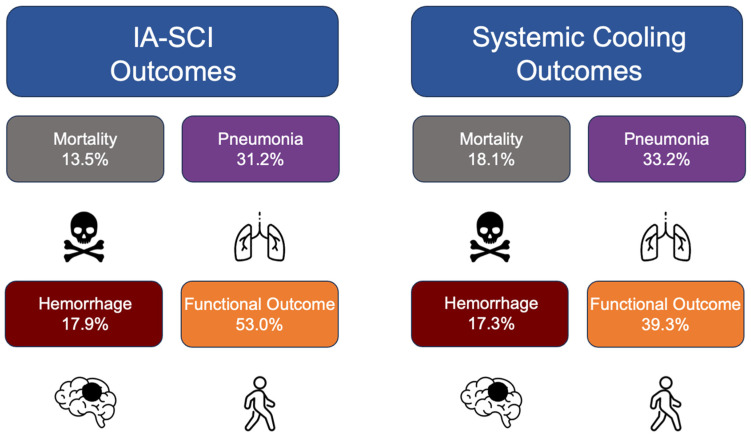
Comparison of mortality, pneumonia, hemorrhages, and functional outcomes between IA-SCI and systemic cooling.

Based on our review, mortality rates were up to 47.0% within the hypothermic groups that were systemically cooled, while mortality rates ranged from 7.0% to 20.0% in IA-SCI groups (Figure 1). The percentage of patients who were functionally independent was higher in both systemic and IA-SCI groups compared with the normothermic groups. Targeted temperature management reduces brain edema, the metabolic rate of brain tissue, and the cascade of cellular events leading to neuronal death, thereby limiting ischemic damage [6,39,40,41,42,43,44,45,46,47,48]. Neugebauer et al. (2019) found that there was no significant difference in mortality between patients treated with moderate hypothermia and those given standard care after an early hemicraniectomy for a malignant MCA stroke [12]. The effectiveness of hypothermia in treating malignant stroke is subject to debate. While hypothermia appears to offer advantages over standard medical treatment, its efficacy is considerably lower than that of a hemicraniectomy [36]. Following the release of successful trial results for hemicraniectomies, many centers have started using hypothermia in conjunction with a hemicraniectomy. Yet, the evidence supporting this combined approach remains limited. While TTM is a feasible treatment for AIS, especially when administered alongside thrombolysis, its benefits in terms of significant functional improvements and reduced mortalities are still uncertain.

Pneumonia rates were higher in patients receiving TTM. The COOL AID study reported noncritical complications such as bradycardia, hypotension, and infections in the hypothermia group, with three deaths occurring during the study [33]. The high rates of serious adverse events observed in some trials may be directly associated with the hypothermic treatment, potentially leading to higher risks with mechanical ventilation due to prolonged sedation during the hypothermic treatment. Wan et al. 2023 reported a statistically significant difference in the occurrence of pneumonia between normothermic and hypothermic test groups [19]. Lyden et al. (2016) also indicated that this protocol, which included drugs known to cause sedation and swallowing reflex impairment, might have contributed to the increased incidence of pneumonia, suggesting aspiration as a potential cause [17]. The application of TTM to patients with strokes could occur due to multiple reasons including a suppressed immune response [67], reduced cough reflex, reduced airway clearance [68], and impaired mucociliary function [69]. However, it remains unclear if pneumonia can be effectively prevented through preemptive antibiotic treatment [70].

Normothermic and hypothermic stroke patients did not differ in their NIHSS values or hemorrhage rates (Figure 1). While TTM does not prevent the initial hemorrhage, it can potentially reduce secondary brain injury and further hemorrhaging through the reduction of cerebral edema [6], vasoconstriction [71], and possible normalization of coagulation effects [72]. Future studies are required to refine hypothermia protocols, determine optimal target temperatures, and establish the most effective durations of therapy to improve clinical outcomes.

### 4.9. Recommendations for TTM in Stroke Patients

**Figure 2:**
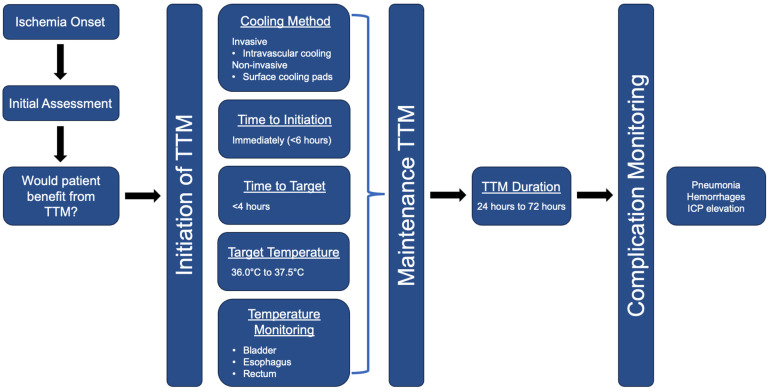
Recommendations for targeted temperature management in stroke patients, including best practices for setting the target temperature, prompt initiation and induction, suitable duration, cooling techniques, and precise temperature monitoring.

The limited available data suggest that adhering to the best practices of target temperature setting, timely initiation and induction, appropriate duration, method of cooling, and accurate temperature monitoring can improve outcomes among stroke patients (Figure 2). While there is no consensus for the optimal target temperature for TTM, maintaining a patient’s core temperature between 36.0 °C and 37.5 °C should be considered [50]. Lowering the body temperature within this range has been shown to reduce metabolic demand and neuronal excitotoxicity, thus protecting the brain from further injury [73]. However, individual patient factors, such as the severity of the stroke and pre-existing conditions, must be taken into account when determining the specific target temperature. Research suggests that the sooner TTM is initiated following a stroke, the better the outcomes for the patient [59]. Ideally, cooling should commence within the first 3–6 h post-stroke as delays may diminish the neuroprotective effects of TTM. Reaching the target temperature with a rapid induction phase, preferably within 4 h, is recommended for maximum efficacy [74]. The rate of cooling should be monitored closely to prevent complications associated with too rapid a decrease in body temperature. The duration of TTM varies based on the stroke’s severity and type but typically ranges from 24 to 72 h. Longer durations may be considered for severe cases, although this can increase the risk of complications like infections and coagulopathies [74]. Various methods are available for inducing hypothermia, including invasive techniques like intravascular cooling and non-invasive methods such as surface cooling pads. While invasive methods offer more precise temperature control, they carry a higher risk of complications. Non-invasive methods are less risky but might not provide the same level of temperature regulation [75]. Common sites for accurate temperature monitoring include the bladder, esophagus, and rectum. The esophageal site is often preferred due to its proximity to the heart and lungs, providing a more accurate reflection of core temperature [76]. Since there are no consensus guidelines for TTM in stroke patients, the continuous research and refinement of TTM protocols are necessary for fully harnessing its benefits.

### 4.10. Limitations and Future Research

This review of the literature is limited by its inability to provide a comprehensive overview of all relevant studies, which may introduce potential biases in our conclusions. In addition, we were unable to evaluate most informational materials, which may lead to an overestimation of the quality of the studies provided. Further limitations of this study include the variation of study designs among trials. Protocol deviations, such as that in the method of systemic cooling, were common even within single studies. Differences in concomitant therapies (i.e., recanalization or hemicraniectomy) may influence the significance of TTM as a stand-alone therapy. Methods of measuring outcome and collection time points were also inconsistent, with some studies using a modified Rankin Scale score and others using a post-treatment mRS score. This may compromise our ability to meaningfully interpret the findings of the studies with regard to the efficacy of TTM. Though feasibility studies have demonstrated the safety of TTM after a stroke, the small sample size of most studies constrained the generalizability of TTM as a beneficial therapy. The insufficient number of patients within these studies often restricted the ability to provide statistically significant findings. This remains an on-going limitation within the field as two large prospective, randomized clinical trials were prematurely terminated due to the slow recruitment of patients within the funding period [9,17].

Given these limitations, future research should focus on standardizing protocols, particularly in the method of TTM application and the measurement of outcomes, to allow for more direct comparisons between studies. There should also be an emphasis on larger-scale studies or meta-analyses to overcome the issue of small sample sizes and improve the generalizability of the results. By investigating combination therapies, such as thrombectomy and TTM, researchers might be able to isolate the specific contributions of TTM in the context of a broader treatment strategy, thereby providing a more nuanced understanding of its efficacy. This direction is not only promising for enhancing stroke care but also aligns with the current trend in medical research towards personalized and combination therapies.

## 5. Conclusions

This literature review provides a comprehensive examination of TTM in the treatment of AIS. Through an exhaustive analysis of the current research, we have identified that TTM, when administered in a timely and effective manner, holds promise for mitigating the impact of stroke-related neuronal injuries. Although TTM can potentially reduce the infarct size and improve neurological outcomes, the optimal application of this therapy remains complex, being influenced by factors such as the time to initiation, target temperature, duration of cooling, and method of cooling. TTM is also associated with challenges such as higher rates of pneumonia and the need for a precise balance between therapeutic benefits and potential risks. The review underscores the need for continued research to refine TTM protocols, particularly regarding the induction phase, target temperature, and duration of treatment, to maximize its benefits in clinical practice. Additionally, we highlight the importance of considering patient-specific factors, such as stroke severity and comorbidities, in the application of TTM. Ultimately, our findings suggest that TTM, despite being a complex intervention, offers a valuable addition to the current arsenal of treatments for AIS and has the potential to significantly improve patient outcomes when used judiciously and in conjunction with other therapeutic strategies.

## Data Availability

Data sharing is not applicable to this article as no new data were created or analyzed in this study.
